# The Essential Role of Vitellogenin Receptor in Ovary Development and Vitellogenin Uptake in *Bactrocera dorsalis* (Hendel)

**DOI:** 10.3390/ijms160818368

**Published:** 2015-08-07

**Authors:** Lin Cong, Wen-Jia Yang, Xuan-Zhao Jiang, Jin-Zhi Niu, Guang-Mao Shen, Chun Ran, Jin-Jun Wang

**Affiliations:** 1 Key Laboratory of Entomology and Pest Control Engineering, College of Plant Protection, Southwest University, Chongqing 400715, China; E-Mails: iamconglin@126.com (L.C.); yangwenjia10@126.com (W.-J.Y.); jxzzby@163.com (X.-Z.J.); jinzhiniu@yahoo.com (J.-Z.N.); blackaet@163.com (G.-M.S.); 2 Citrus Research Institute, Southwest University, Chongqing 400712, China; E-Mail: ranchun@cric.cn; 3 College of Biology and Environmental Engineering, Guiyang University, Guiyang 550005, China

**Keywords:** *Bactrocera dorsalis*, vitellogenin receptor, yolk protein, ovary, RNA interference

## Abstract

The vitellogenin receptor (VgR) functions as an essential component in uptaking and transporting vitellogenin (Vg) in female adults, which is involved in ovary development and oviposition. This study aimed to clarify the molecular characteristics and function of VgR in the oriental fruit fly *Bactrocera dorsalis* (Hendel). Here, we identified the full-length of *BdVgR* (GenBank Accession No. JX469118), encoding a 1925 residue (aa) protein with a 214.72 kDa molecular mass and several typical motifs of low-density lipoprotein receptor superfamily (LDLR). Phylogenic analysis suggested that *Bd*VgR was evolutionary conserved with other Dipteran VgRs. The expression of *BdVgR* was exclusively detected in the ovaries rather than head, thorax or other tissues. The developmental expression patterns showed that the signal of *BdVgR* was detectable in very beginning of adult stage, and positively correlated with the growth rate of ovaries and the expression levels of its ligands. In addition, we also demonstrated that the expression level of *BdVgR*, and ovary development were significantly suppressed after being injected with *BdVgR-*targeted dsRNA. Together, all of these results indicated that *Bd*VgR was critical for yolk protein absorption and ovary maturation in *B. dorsalis*, playing a vital role in female reproduction.

## 1. Introduction

In insects, including oviparous species, successful reproduction is fundamental in maintaining their population, which relies on two key indispensable steps: vitellogenin (Vg) biosynthesis and deposition. After being synthesized in the fat body and released into the hemolymph, Vg is taken up into the developing ovaries via endocytosis by its receptor, vitellogenin receptor (VgR), which is located on the surface of the oocytes within clathrin-coated pits [1]. Then, this lipoprotein is transported to its target recipient cell by VgR, providing multiple nutritional elements to support the developments of oocytes [2].

It is documented that only a single VgR gene exists in most insect species, which could generate a long transcript, about 7.5 kb, and encode an ovary-specific polypeptide with a molecular weight of 180–214 kDa, approximately twice the VgR in vertebrates [1]. *In silico* analysis further suggests that insect VgR is a member of the low density lipoprotein receptor (LDLR) superfamily, which is characterized by five highly conserved, but functionally different, amino acids domains: the ligand-binding domain (LBD) containing several Class A cysteine-rich repeats (LDLR_A_), the epidermal growth factor (EGF)-precursor domain comprising Class B cysteine-rich (LDLR_B_, EGF-like repeats) and YWXD repeats, the O-linked sugar domain, the transmembrane domain, and the cytoplasmic tail domain with internalization signals [3].

The molecular characteristics of VgRs are not only identified in invertebrates, like insects [3], mites [4], ticks [5,6,7], shrimps [8,9,10], crabs [11], and nematodes [12], but also in vertebrates, such as fish [13,14,15,16], frogs [17] and chickens [18]. However, only five VgRs have been identified in Dipteran insects, including *Aedes aegypti* [19], *Anopheles gambiae* [20], *Ceratitis capitata* (genomic sequence), *Drosophila melanogaster* [21] and* Musca domestica* (genomic sequence). Therefore, there is not enough available information to get the full understanding of the reproductive mechanism of this order, considering its economic importance.

The oriental fruit fly,* Bactrocera dorsalis* (Hendel), is one of the most devastating pests of fruits and vegetables throughout some areas of Asia and the Pacific, causing grave economic losses [22]. Unfortunately, there is still lack of effective strategies to manage this pest, owing to its unique biological properties, such as the rapidly increased insecticide resistance, high fecundity and oviposition traits [22,23,24]. Although, the yolk protein genes of this species, *Bdyp1* and *Bdyp2*, have been well investigated in previous study [25], the information on their receptor is still lack. Thus, illustrating the function of VgR in *B. dorsalis* will help obtain a more comprehensive understanding of the process of reproduction, and eventually develop new strategies to control this pest.

In this research, we identified the full-length of VgR from *B. dorsalis* (*BdVgR*), analyzed and compared the basic molecular and structural characteristics with those from other insects. In addition, we reported the spatial- and temporal-expression pattern of *BdVgR* by semi-quantitative PCR and quantitative PCR (*q*PCR), along with the expression profiles of its ligands (*Bdyp1* and *Bdyp2*) and ovarian development in time course. Finally, we further verified the function of *Bd*VgR by silencing its expression by RNA interference (RNAi).

## 2. Results

### 2.1. Sequence and Structural Characteristics of VgR in B. dorsalis

The cDNA sequence of *BdVgR* (GenBank Accession No. JX469118) was generated from Seven-day-old female adult of the oriental fruit fly. The full-length was 6595 bp, consisting of a 290-bp 5′-untranslated region (UTR) and a 530-bp 3′-UTR, a putative open reading frame (ORF) of 5775 bp, in addition with a polyadenylation signal “AATAAA” at 481 bp downstream of the stop codon.

**Figure 1:**
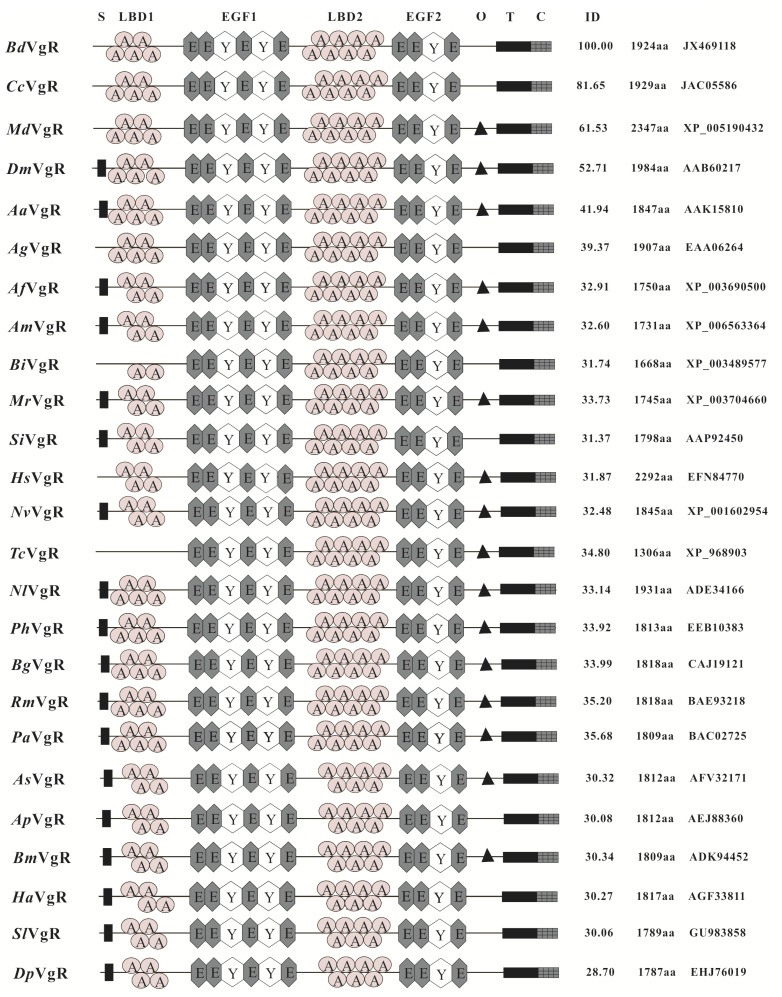
Diagrammatic comparison of typical domains of *B. dorsalis* vitellogenin receptor with other insect vitellogenin receptor. S, signal peptide; LBD, ligand binding domain; EGF, EGF-precursor domain; O, O-linked sugar domain; T, transmembrane domain; C, cytoplasmic domain; ID, identity; A, LDLR_A_; E, EGF-like repeat; Y, YWTD; *Bd*VgR, *Bactrocera dorsalis*, JX469118; *Cc*VgR, *Ceratitis captitata*, JAC05586; *Md*VgR, *Musca domestica*, XP_005190432; *Dm*VgR, *Drosophila melanogaster*, AAB60217; *Aa*VgR, *Aedes aegypti*, AAK15810; *Ag*VgR, *Anopheles gambiae*, EAA06264; *Af*VgR, *Apis florea*, XP_003690500; *Am*VgR, *A. mellifera*, XP_006563364; *Bi*VgR, *Bombus impatiens*, XP_003489577; *Mr*VgR, *Megachile rotundata*, XP_003704660; *Si*VgR, *Solenopsis invicta*, AAP92450; *Hs*VgR, *Harpegnathos saltator*, EFN84770; *Nv*VgR, *Nasonia vitripennis*, XP_001602954; *Tc*VgR, *Tribolium castaneum*, XP_968903; *Nl*VgR, *Nilaparvata lugens*, ADE34166; *Ph*VgR, *Pediculus humanus corporis*, EEB10383; *Bg*VgR, *Blattela germanica*, CAJ 19121; *Rm*VgR, *Rhyparobia maderae*, BAE93218; *Pa*VgR, *Periplaneta americana*, BAC02725; *As*VgR: *Actias selene*, AFV32171; *Ap*VgR, *Antheraea pernyi*, AEJ88360; *Bm*VgR, *Bombyx mori*, ADK94452; *Ha*VgR, *Helicoverpa armigera*, AGF33811;* Sl*VgR, *Spodoptera litura*, GU983858;* Dp*VgR, *Danaus plexippus*, EHJ76019.

The ORF of *BdVgR* encoded a 1925 amino acid sequence, with the predicted molecular weight (*M*w) of 214.72 kDa and the theoretical isoelectric point (pI) of 5.89. Analysis of *Bd*VgR revealed that no signal peptide was identified in the N-terminal of the putative protein sequence (Figure 1). Based on the results of conserved domain analysis, *Bd*VgR was supposed to be a member of the low-density lipoprotein receptor (LDLR) superfamily, containing four classical and conserved modules, such as ligand-binding domain (LBD), epidermal growth factor (EGF) precursor domain, transmembrane domain and cytoplasmic domain. In total, *Bd*VgR possessed thirteen LDLR class A repeats (LDLR_A_) located in the two LBDs, with five and eight LDLR_A_ repeats in the first and second LBD domain, respectively. Both of the LDBs were followed by an EGF-precursor domain. In addition, calcium-binding EGF-like domain, YWTD domain and LDLR class B repeats (LDLR_B_) were all identified in both of the EGF-precursor domains. According to the prediction of TMHMM server, the hydrophobic transmembrane domain was located at 1766–1788 aa, followed by the cytoplasmic domain at 1789–1924 aa with one di-leucine LL internalization signal motif (1834–1835 aa). However, O-linked sugar domain was not identified in *Bd*VgR.

### 2.2. Sequences Comparison and Phylogenetic Analysis

**Figure 2:**
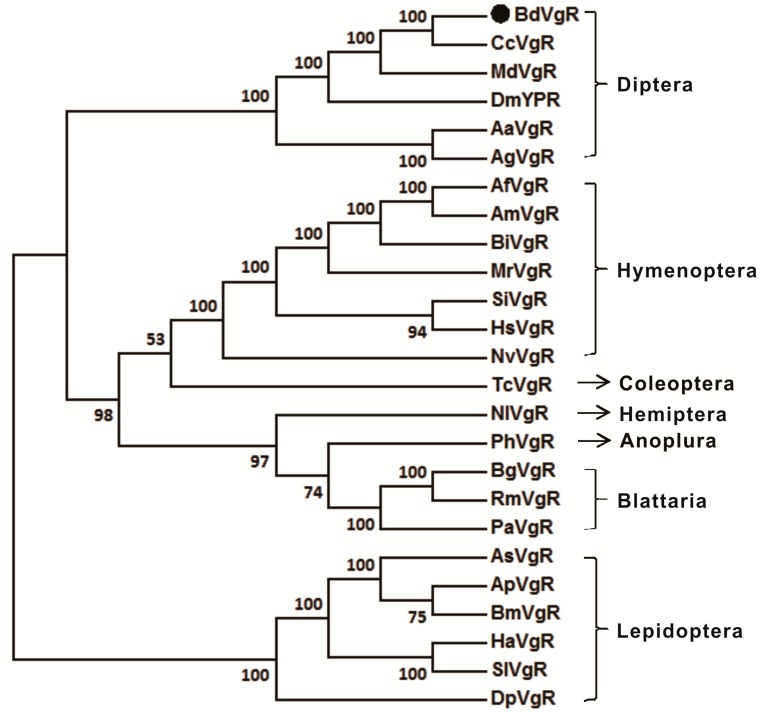
A phylogenetic tree of vitellogenin receptors. The amino acid sequences of *Bd*VgR 24 and other insect VgR sequences were selected to analyze the evolutionary relationship using the Neighbor-Joining method with a bootstrap value of 1000. The dot stands for protein sequence of VgR from *B. dorsalis*.

The comparison of *Bd*VgR with other insect VgRs indicated that the amino acid sequences had a similar structural feature, especially within the sequences from the same insect order (Figure 1). However, the total number of LDLR_A_ repeats varied among different insect orders. The *Bd*VgR sequence had a Dipteran typical 13 LDLR_A_ repeats, whereas there were 11, 12 (10 for *Bombus impatiens*), 13, 13 and 8 LDLR_A_ repeats in Lepidoptera, Hymenoptera, Blattaria, Homoptera and Coleoptera, respectively. Besides, the O-linked sugar domains were not conserved even in the same insect order (except for Blattaria). In addition, the multiple alignments showed that *Bd*VgR shared high identity with the VgRs from Dipteran species. The identities were 41.94%, 39.37%, 81.65%, 52.71% and 61.53%, comparing to the *Bd*VgR sequence to those generated from *A. aegypti* [19], *A.gambiae*, *C. capitata*, *D. melanogaster* [21] and *M*. *domestica*, respectively. However, the identities were much lower when comparing *Bd*VgR with other insect VgRs. In addition, the phylogenetic tree suggested that *Bd*VgR shared a closer ancestry relationship with *Cc*VgR compared with *Md*VgR and *Dm*YPR. As expected, the VgRs from Dipteran were clustered into a separate clade from other insects (Figure 2).

### 2.3. Tissue-Specific Expression Pattern of BdVgR

**Figure 3:**
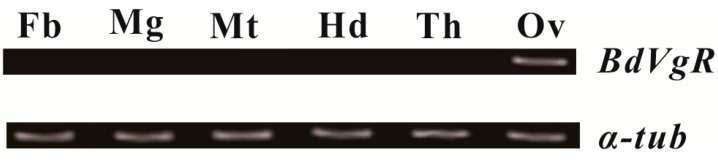
Spatial-expression patterns of *BdVgR* in different body segments and tissues of seven-day-old female adults. The tissues and segment included the fat body (Fb), midgut (Mg), Malpighian tubule (Mt), head (Hd), thorax (Th), ovary (Ov). And *α-tubulin* (*α-tub*) was used as a reference gene. The thermal cycles for PCR amplification was 30 times, yielding 163 and 184 bp PCR products for *BdVgR* and *α-tubulin*, respectively.

To elucidate the tissue-specific patterns of *BdVgR*, semi-quantitative PCR was-conducted to detected its expression abundance among different segments and tissues from seven-day-old female adults. As a positive control, *α-tubulin* was detected in all segments and tissues. However, the expression levels of *BdVgR* were quite different among those samples. Apparently, *BdVgR* was expected to be expressed in the ovaries exclusively (Figure 3).

### 2.4. Ovary Growth, Developmental-Specific Expression Patterns of BdVgR, Bdyp1 and Bdyp2

**Figure 4:**
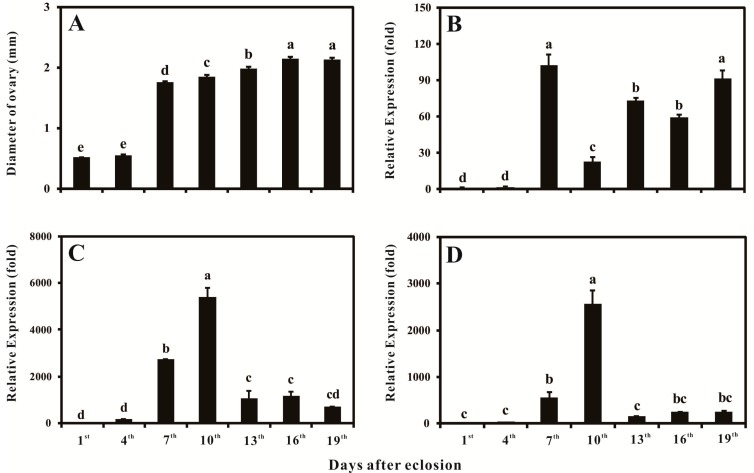
The growth of ovary and temporal-expression patterns of *BdVgR*,* Bdyp1* and* Bdyp2*: (**A**) the developmental status of ovaries; (**B**) the temporal-expression pattern of *BdVgR*; (**C**) the temporal-expression pattern of *Bdyp1*; (**D**) the temporal-expression pattern of *Bdyp2*. The bars represented the mean ± SE. Different letters indicated significant difference with ANOVA (Least Significant Difference, LSD, *p* < 0.05).

To evaluate the potential relationship between the development of ovary and *BdVgR*, the diameters of ovaries and the expression profiles of *BdVgR* were monitored throughout the different ages of female adults, in addition with its ligands, *Bdyp1* and *Bdyp2* (Figure 4). As demonstrated in Figure 4A, no obvious enlargements were detected during the first four days. However, a dramatic enlargement was observed on the seventh day, kept increasing in the following days, and finally reached the maximum size on the 16th day (2.146 ± 0.0354 mm), which was 4.13 times larger than that on the first day (0.522 ± 0.0064 mm). The developmental expression patterns showed that the mRNA of *BdVgR*, *Bdyp1* and *Bdyp2* were detectable throughout all selected time points, but extremely low in the very beginning of the adult stage (Figure 4B–D). Synchronous with the dramatic change in ovarian development, the expression level of *BdVgR* was also significantly increased to its summit on the seventh day, which was 102.31 times of that in the first day adults (Figure 4B). However, there was a turning point on the tenth day. The expression level of *BdVgR* quickly down-regulated, decreasing to 21.85% compared to the expression level of the seventh day, but finally recovered to a certain high level during the following days. Interestingly, *Bdyp1* and *Bdyp2* had similar transcriptional patterns, as they started to increase steeply in the seventh day and reached their maximum levels in the tenth day. However, the highest expression level of *BdVgR* appeared on the seventh day, three days earlier than that in *Bdyp1* and *Bdyp2* (Figure 4C,D).

### 2.5. Silencing BdVgR Expression by RNA Interference

**Figure 5:**
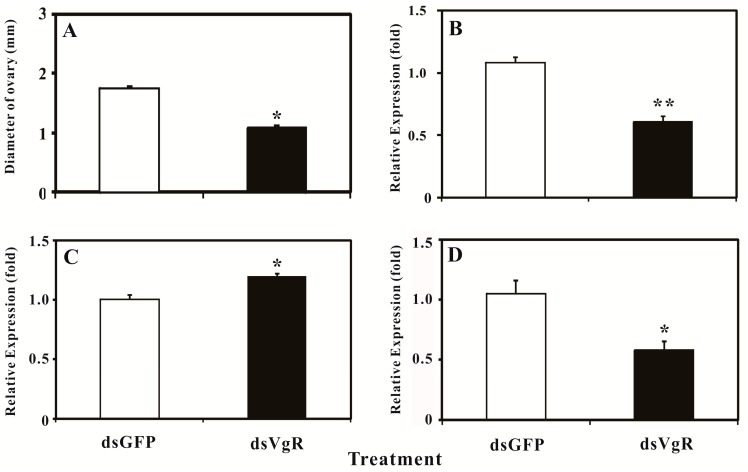
The ovary diameter and expression levels of *BdVgR*, *Bdyp1* and *Bdyp2* 72 h after injected with dsRNA. (**A**) The diameters of ovaries; (**B**) The mRNA level of *BdVgR*; (**C**) The mRNA level of *Bdyp1*; (**D**) The mRNA level of *Bdyp2*. The bars represent the mean ± SE. The asterisks indicate significant difference with *t*-test, * *p* < 0.05 or ** *p* < 0.01.

To verify the potential role of *Bd*VgR in yolk protein uptake and ovary development, *BdVgR*-targeted dsRNA was synthesized and injected into the four-day-old female adults. The RNAi effects were determined by ovarian dissection and *q*PCR on the third day after injection, with the indicators of ovarian diameter and expression levels. Obviously, the development of ovaries was significantly arrested in dsBdVgR-treated group (Figure 5A). The diameters of ovaries dramatically reduced (1.084 ± 0.0361 mm), ranging from 0.760 to 1.483 mm (*p* = 0.039). Conversely, the diameter of ovaries from the dsGFP-treated group (1.751 ± 0.0243 mm), ranging from 1.490 to 1.927 mm, showed no significant difference with the seven-day-old control insects (1.761 ± 0.0206 mm, *p* =0.37). Coincident with the decreased size of ovaries, there was a significantly reduced *BdVgR* transcript level in female adults (43.7%) derived from dsBdVgR-treated group (*p* = 0.002) (Figure 5B). In addition, the expression profile of *Bdyp1* was up-regulated in the dsBdVgR treated group (*p* = 0.010) (Figure 5C), and *Bdyp2* was down-regulated (*p* = 0.021) (Figure 5D).

## 3. Discussion

Here, the complete VgR sequence was obtained from *B. dorsalis*. This was the first report of VgR from a *Tephritidae* to date. As expected, *Bd*VgR harbored several typical functional motifs of VgRs and the LDLR superfamily, namely, ligand binding domains, EGF-precursor domains, transmembrane domain and cytoplasmic tail domain, and showed similarity to the sequences (VgRs and YPR) in insect, particularly to those sequences in Diptera, perhaps due to the evolutionary conservation in their ligands (Figure 1). Although those motifs are conserved in insect, there were some special traits in *Bd*VgR sequence. Similar to the VgR sequences in Diptera and Blattaria, *Bd*VgR also had a typical 13 cysteine-rich LDLR_A_ repeats with classical arrangement, which was five and eight repeats in the first and second LBD, respectively [26,27,28]. However, the number of LDLR_A_ repeats and arrangement are quite different from other insect orders and species. For example, there are four- and seven-LDLR_A_ repeats in Lepidoptera, four/two and eight repeats in Hymenoptera, single eight-repeats in Coleoptera (*Tribolium castaneum*), and four and eight repeats in ticks [5,29] and vertebrates [9]. In addition, both of the EGF-precursor domains contained a calcium-binding site. However, no O-link sugar domain appeared in the C-terminal of *Bd*VgR sequence, although it was supposed to play a role in improving VgR stability and regulating the signal pathway [2] and very conserved in Blattaria [26,28] and present in *A*. *aegypti* [19], *D*. *melanogaster* [21], *M*. *domestica* and other insects. Besides, only one LL motif was found in the cytoplasmic tail of *Bd*VgR, and no NPXY motif was found as described in the fire ant [30] and cockroaches [26,27,28].

Unlike its ligands, which are widely detected in the fat body, ovary, head and thorax in the female adult [25], the *BdVgR* mRNA were exclusively expressed in ovary and no traces were detected in the other tissues or segments (Figure 3). The result is along with the reports in other insect and vertebrates, such as *D. melanogaster* [21], *A. aegypti* [19], *Bombyx mori* [31], *Antheraea pernyi* [32], *Spodoptera litura* [33], *Periplaneta americana* [28], *Leucophaea maderae* [26], *Solenopsis invicta* [30], *Thunnus thynnus* [34] and *Oncorhyn chusmykiss* [35], consistent with its specific distribution and conserved role for egg development. However, recent studies point out that the VgR is no longer exclusively located in ovaries of female adults: the fat body [32], hypopharyngeal glands [36], head and even embryos [37] are found to be the extraovarian tissues or segment that express *VgR*; this may due to the alternative functions of their ligands in food storage [37], immunity [38] and resistance [39].

The relationship between expression level of VgR and ovarian development has been extensively studied in a variety of insects, such as, cockroach [26,27,28], fire ant [30], armyworm [33] and vertebrates, like shrimp [9], trout [35] and chicken [40]. The developmental-specific analysis indicated that the *BdVgR* shared a similar mRNA expression pattern with other insects, which expressed at all ovarian developmental stages, extremely low in the very beginning of previtellogenic stage, increased dramatically before fully vitellogenic period. To be more exact, the transcript pattern of *BdVgR* was up-regulated with the process of sexual maturation (Day 1–7), but quick declined when the ovaries fully developed and oviposition initiated (Day 9–10) (Figure 4), in coordination with the expressions of *Bdyp1* and *Bdyp2*. The results demonstrated that the enlargement of ovary depends on vitellogenesis and endocytosis process. The over-expressed *BdVgR* may work as a precondition for Vgs to endocytosis effectively in female adults. Interestingly, comparing the high expression level of *BdVgR*, the transcript levels of *Bdyp1* and *Bdyp2* were relative low from the tenth day (after egg-laying), which may indicate that other ligands rather than yolk proteins play a more important role thereafter.

RNAi is now used as a powerful tool in gene functional study in biology. Therefore, we conducted an RNAi experiment to verify the function of *BdVgR* in the female adults. Considering the developmental expression pattern and the persistence of the knock-down effect of RNAi in *B. dorsalis* [41,42,43], four-day-old females and 0.7 μg exogenous dsRNA per insect were selected as the appropriate stage and the optimal dose for microinjection based on the preliminary trials. Clearly, an effective gene silence was achieved, causing a significant reduction in mRNA expression of *BdVgR* and inhibition of ovarian size and development (Figure 5A,B), indicating its role in transporting the nutrients into the growing oocytes and egg development. The similar results are also found in cockroach [27], silkworm [31], armyworm [33], fire ant [44] and tick [7]. Besides gene suppression and ovarian developmental delay, egg formation failure, less egg laying, oviposition delay, and accumulation of Vgs are also regarded as the equivalent phenotypes caused by VgR knockdown. The expressions of *Bdyp1* and* Bdyp2* have also have been influenced (Figure 5C,D). The transcript level of *Bdyp1* was up-regulated and *Bdyp2* was down-regulated in the dsBdVgR-treated group, this may be owing to the different regulation mechanisms of the two genes in this species [25]. Taken together, all of those results demonstrated *BdVgR*’s key role in transporting yolk protein and ovary development in female adults of the oriental fruit fly.

## 4. Experimental Section

### 4.1. Insects Rearing, Sample Preparation and Ovarian Diameter Measurement

The oriental fruit flies were maintained in the laboratory under 27 ± 1 °C, 70%–80% relative humidity, 14:10 h (L:D) photoperiod conditions and reared as described in our previous study [45]. Sufficient newly emerged (1-day-old) virgin females were selected randomly and maintained in the same controlled condition. The females were collected at 3-day intervals from the first day after eclosion to the following nineteen days.

Tissues (including the fat body, midgut, Malpighian tubules and ovaries) and body segments (heads and thoraxes) were dissected from 7-day-old females in Ringer’s saline under a binocular stereoscope (Olympus SZX12, Tokyo, Japan). Tissue and body segment samples were all kept in RNAstore Reagent (Tiangen Biotech, Beijing, China) and stored at −80 °C until RNA isolation.

Fifteen female adults from different time points were randomly collected and carefully dissected for ovarian diameter measurement. The diameters of the ovaries were measured with an M165C microscope equipped with LAS v3.7 (Leica Microsystems, Wetzlar, Germany). Statistical difference of the ovarian diameter was analyzed with ANOVA by SAS 8.01 program (SAS Institute Inc., Cary, NC, USA) and reported as mean ± SE (LSD or *t*-test, *p* < 0.05).

### 4.2. RNA Extraction and Cloning of BdVgR

Total RNA from different tissues was isolated with RNeasy^®^ Plus Mini Kit (Qiagen GmbH, Hilden, Germany). Total RNA from the whole flies and segments was extracted with TRIzol reagent (Invitrogen, Carlsbad, CA, USA) and then treated with RQ1 RNase-Free DNase (Promega, Madison, WI, USA) to eliminate genomic DNA contamination. All of the RNA samples were detected with agarose gel electrophoresis and measured with NanoVue spectrophotometer (GE Healthcare Bio-Science, Uppsala, Sweden) to ensure the integrity, quality and concentration. The PrimeScript™ RT reagent Kit (TaKaRa, Dalian, China) was applied to synthesize first strand cDNA with 0.5 μg total RNA. All manipulations were followed the manufacturer’s protocols.

Based on the transcriptome data of *B. dorsalis* [46], four pairs of gene-specific primers (*BdVgR*-F/R1-4, Table S1) were designed to amplify the fragments of *BdVgR*. Further, another two pairs of gene-specific primers (*BdVgR-*3R1/2, *BdVgR-*5R1/2, Table S1) were designed to determine the 5′- and 3′-flanking regions of *BdVgR* using SMARTer™ RACE cDNA Amplification Kit (Clontech, Mountain View, CA, USA). All gene-specific primers were examined by DNAMAN 5.2.2 (Lynnon, PQ, Canada) to check their self-complementarities and melting temperatures.

The 25 μL PCR reaction system included: 1.0 μL of cDNA, 1.0 μL of forward and backward primer (10 μM), 2.0 μL of dNTPs (2.5 mM each), 2.5 μL of Mg^2+^ (25 mM), 2.5 μL of 10× PCR buffer (Mg^2+^ free), 15.0 μL nuclease-free water and 0.25 μL of rTaq (5 U/μL) (Takara). All the PCR reactions were conducted as the following thermal cycles: 3 min at 94 °C, 33 cycles of 30 s at 94 °C, 30 s at 55–58 °C, 2 min at 72 °C, and final extension at 72 °C for 10 min. The purified PCR products were cloned into the pGEM^®^-T Easy vector (Promega, Madison, WI, USA), transformed into DH5α competent cells (Biomed, Beijing, China) and finally sequenced (Invitrogen, Shanghai, China).

### 4.3. Bioinformatics Analysis of BdVgR

SignalP 4.1 was used to predict the putative signal peptide (http://www.cbs.dtu.dk/services/SignalP/) [47]. DNAMAN 5.2.2 was adopted to assemble the full-length of cDNA, predict the molecular weight and isoelectric point of the deduced polypeptide. The conserved motifs were analyzed with the Conserved Domain Database in NCBI (http://www.ncbi.nlm.nih.gov/cdd/) [48] and SMART database (http://smart.embl.de/) [49]. The GPP prediction server was applied to predict the O-linked sugar domain (http://comp.chem.nottingham.ac.uk/glyco/) [50]. The transmembrane region was predicted by the TMHMM Server v.2.0 (http://www.cbs.dtu.dk/services/TMHMM/). MEGA 5.04 was applied to construct the phylogenetic tree using the Neighbor-Joining method with a bootstrap value of 1000 [51].

### 4.4. Semi-Quantitative PCR and Quantitative PCR (qPCR)

Semi-quantitative PCR was performed to compare the transcript abundance of *BdVgR* in different tissues and segments with gene-specific primers (*BdVgR*-qF/R) (Table S2), using *α-tubulin* (*α-tubulin-*qF/R, GenBank Accession number: GU269902, Table S2) as an internal control [52]. The PCR amplification condition was 95 °C for 3 min and 30 cycles of 95 °C for 30 s, 60 °C for 30 s, 72 °C for 30 s, and 72 °C for 5 min for final extension. The 25 μL PCR reaction system included: 1.0 μL of cDNA, 1.0 μL of forward and backward primer (5 μM), 2.0 μL of dNTPs (2.5 mM each), 2.5 μL of Mg^2+^ (25 mM), 2.5 μL of 10× PCR buffer (Mg^2+^ free), 15.0 μL nuclease-free water and 0.25 μL of rTaq (5 U/μL) (Takara).

*q*PCR was further applied to investigate the transcript levels of *BdVgR*, *Bdyp1* (GenBank Accession No. AF368053) and *Bdyp2* (GenBank Accession No. AF368054) at different developmental stages of female adults, with gene-specific primers (*BdVgR*-qF/R, *Bdyp1*-qF/R and *Bdyp1*-qF/R, Table S2) and *α-tubulin* (*α-tubulin-*qF/R, Table S2) as an internal control. The *q*PCR reaction was performed in the StepOne Plus Real-Time PCR System (ABI, Carlsbad, CA, USA) with 1.0 μL cDNA, 1.0 μL forward and backward primer (10 μM), 7.0 μL GoTaq^®^ *q*PCR Master Mix (Promega) and 10.0 μL nuclease-free ddH_2_O. The reaction condition was: 95 °C for 2 min, 40 cycles of 95 °C for 15 s and 60 °C for 30 s, followed by melting curve analysis. The relative mRNA levels were determined using the 2^−ΔΔ*C*t^ method [53]. Three independent replications were carried out for each reaction sample. The statistical analysis and data were conducted and presented as described above (SAS, *p* < 0.05).

### 4.5. RNA Interference

To obtain the *BdVgR*-targeting dsRNA, dsVgR, a 558 bp fragment (include T7 promoter region) located at the LBD (from 1012 to 1184 aa) was synthesized with the primers *BdVgR*-dsTF/TR (Table S2) as in previous study and finally eluted in DEPC water [41]. To examine the RNA interference effect of *BdVgR*, a total amount of 0.7 μg dsVgR was injected into the ventral abdomen of each 4-day-old virgin female adult using Nanoject II Auto-Nanoliter Inject (Drummond Scientific, Broomall, PA, USA). In addition, the dsRNA of GFP, dsGFP (Table S2), was also synthesized and injected as a negative control. After injection, all adults were removed and maintained in the same controlled condition and supplied with artificial diet. Sixty insects were treated in each group, and the experiments were independently repeated for three times. After 72 h, 6 female adults from each group (dsVgR and dsGFP) were selected randomly for RNA extraction and transcript level detection with *q*PCR. Besides, fifteen female adults were harvested for ovary dissection and ovarian diameter measurement. The expression levels and ovarian diameter were evaluated and reported as described above. The *t*-test was employed to evaluate the differences between these two groups (SAS, *p* < 0.05 or 0.01).

## 5. Conclusions

In summary, our findings showed that the full-length of *VgR* was obtained and identified from the oriental fruit fly. The molecular characteristics, expression patterns and function of *Bd*VgR indicated its typical conserved molecular structure and essential function in yolk protein transportation and ovary development. Obviously, this research deepened our understanding of the mechanism of reproduction of *B. dorsalis*, providing a potential target in pest control. However, the hormone regulatory mechanism, for example, ecdysone, juvenile hormone and insulin are still unexplained. Thus, further studies will focus on this aspect, aiming to point out which hormone is the most important in regulating reproductive process.

## Supplementary Materials

Supplementary materials can be found at http://www.mdpi.com/1422-0067/16/08/18368/s1.
