# Supplementary material for: The Essential Role of Vitellogenin Receptor in Ovary Development and Vitellogenin Uptake in Bactrocera dorsalis (Hendel)
Source: Int J Mol Sci. 2015 Aug 7;16(8):18368–83. doi: 10.3390/ijms160818368 (PMC4581250; doi:10.3390/ijms160818368)
Supplement: Supplementary file 1 [file ijms-16-18368-s001.pdf]

Supplementary Information

Table S1. Primers used for amplification of the sequence of *BdVgR*.

Primer	Sequence (5'-3')	Function
BdVgR-F1	GGACAACTCGGACGAAATGAAC	Fragment cDNA cloning
BdVgR-R1	GCTACCATCGGCTTCAGACCC	
BdVgR-F2	GTGGACAAATGTTCTGGACG	
BdVgR-R2	AAATGCGTGGGTATGTGG	
BdVgR-F3	TGGCAGTGATACCAGAAGAG	
BdVgR-R3	CCATCGTGACAAGCGAAATC	
BdVgR-F4	GATGGTTCCGATGAGTTG	
BdVgR-R4	CTGCCCGTTTTGTAGTCC	
BdVgR-3R1	TACAAAACGGGCAGCAAA	3′-RACE of *BdVgR*
BdVgR-3R2	GTTACAATCATTTTGGTGCT	
BdVgR-5R1	TGACAGTCGCTCTTGCCG	5′-RACE of *BdVgR*
BdVgR-5R2	GTGCCGATGTCACCGCTC	

Table S2. Primers used for *q*PCR and dsRNA synthesis.

Primer	Sequence (5'-3')	GenBank NO.
BdVgR-qF	CTTGCAAGCGTTGGACTACA	JX469118
BdVgR-qR	CCGAAGAGTTGACAATGCAC	
Bdyp1-qF	CCAGGCGCTACCAATGTAAT	AF368053
Bdyp1-qR	CTTGCCGAAAGGACTCTTTG	
Bdyp2-qF	ATAAGCGCTGGACTGGTCAT	AF368054
Bdyp2-qR	AGGCTGGACCATTGACGTAG	
α-tubulin-qF	CGCATTCATGGTTGATAACG	GU269902
α-tubulin-qR	GGGCACCAAGTTAGTCTGGA	
BdVgR-dsTF	TAATACGACTCACTATAGGG<br>ATGGTTCCGATGAGTTGG	JX469118
BdVgR-dsTR	TAATACGACTCACTATAGGG<br>TCCATCACAAACGAGACG	
GFP-dsTF	TAATACGACTCACTATAGGG<br>CAGTTCTTGTTGAATTAGATG	CAA58789
GFP-dsTR	TAATACGACTCACTATAGGG<br>TTTGGTTTGTCTCCCATGATG	

The underlined parts stand for T7 promoter sequences.
